# Appendicular Skeletal Muscle Index Is Positively Related to Mediterranean Diet Adherence in University Students

**DOI:** 10.3390/diseases13010003

**Published:** 2024-12-28

**Authors:** Paraskevi Detopoulou, Olga Magni, Ioanna Pylarinou, Despoina Levidi, Vassilios Dedes, Milia Tzoutzou, Konstantina Argyri, Evangelia Fappa, Aristea Gioxari, George Panoutsopoulos

**Affiliations:** Department of Nutritional Sciences and Dietetics, University of the Peloponnese, 24100 Kalamata, Greece; p.detopoulou@uop.gr (P.D.); dp4422319@hua.gr (O.M.); nds19124@go.uop.gr (I.P.); v.dedes@go.uop.gr (V.D.);

**Keywords:** skeletal muscle, Mediterranean diet, university students, appendicular skeletal muscle mass index

## Abstract

Skeletal muscle is associated with cardiometabolic health. The appendicular skeletal muscle index (aSMI) represents the skeletal muscle mass “corrected” for height and constitutes a clinically applicable feature of sarcopenia. The relation of the aSMI with dietary habits is not clear, especially in young adults. The present pilot study aimed to investigate the relationship between the aSMI and Mediterranean diet adherence in young adults. A cross-sectional study of 151 university students was conducted. Anthropometry and bioimpedance analysis (TANITA-MC780) were performed. The aSMI was calculated as the sum of the upper and lower extremity muscle masses divided by height squared. The HPAQ questionnaire was used for physical activity evaluation. A validated food frequency questionnaire was used for dietary assessment, and the Mediterranean Diet Score (MedDietScore) was calculated. Multivariate linear regression models with log-aSMI as a dependent variable were applied. The MedDietScore was positively related to log-aSMI in the total sample (B = 0.009, SE = 0.004, *p* = 0.05, R^2^ for total model = 71%) irrespective of gender, age, ΒΜΙ, smoking, physical activity, and academic year and in males (B = 0.027, SE = 0.011, *p* = 0.023 R^2^ for total model = 37%) irrespective οf age and ΒΜΙ. The positive association of the MedDietScore with aSMI supports the importance of the adoption of a Mediterranean dietary pattern—rich in plant proteins and antioxidant/anti-inflammatory compounds—in maintaining/attaining muscle mass in young subjects.

## 1. Introduction

Skeletal muscle is considered a metabolically active organ related to cardiovascular [1] and metabolic health [2]. Skeletal muscle dysfunction may also be associated with inflammatory indices in subjects with obesity [3], as well as in healthy individuals [4]. Under normal conditions, during muscular contractions, muscle secretes myokines that act on muscle, adipose tissue, liver, and brain [5]. However, in cases of skeletal muscle disuse, cytokines and inflammatory molecules are released, such as interleukin 6 (IL-6) and TNF-α, which may increase oxidative stress and muscle atrophy [6,7]. In the long term, a loss of muscle function and mass (sarcopenia) may occur in healthy and diseased states [8,9].

Interestingly, according to recent data, sarcopenia can also occur in young people, mainly related to physical inactivity, inflammatory diseases, nutrient deficiency, metabolic syndrome, various neuromuscular diseases, and heredity [10]. The appendicular skeletal muscle index (aSMI) represents the skeletal muscle mass “corrected” for height, and it is calculated as the ratio of skeletal muscle mass divided by height squared. In detail, the formula for its calculation is as follows: (muscle mass of arms + muscle mass of legs)/height^2^, and the measurement unit is kg/m^2^. The aSMI constitutes a clinically applicable feature of sarcopenia in the criteria of both European [11] and Asian working groups [12]. In addition, low values of the aSMI have been recently related to pre-diabetes [13], hyperuricemia [14], the risk of carotid plaque development [15], and cancer prognosis [16], among others.

In addition to resistance training [17], nutrition has a key role in the development and maintenance of muscle tissue. Studies in people with sarcopenia indicate that the key nutrients beneficial for muscle tissue are mainly vitamin D, calcium, selenium, magnesium, omega-3 fatty acids, and various antioxidants such as vitamin C, vitamin A, and vitamin E [18]. With regard to proteins, the intake of high-biological value proteins and amino acids seems to be a prerequisite for the maintenance and growth of muscle fibers, as these components stimulate protein synthesis [8]. Among recognized dietary patterns, the Mediterranean diet, which is rich in fruits, vegetables, legumes, olive oil, fish, and whole grains, may maintain lean body mass and increase functionality, even in elderly populations [18]. According to previous work, the adoption of the Mediterranean diet relates to better gait, body support, hand strength, and a lower risk of disability [19,20]. So far, data on the relationship between sarcopenia and the Mediterranean diet are scarce and inconsistent [21,22,23]. In a systematic review of seven prospective studies assessing the relationship of the Mediterranean diet to sarcopenia with 253–2948 participants, conflicting results were reported [24], while studies with a long follow-up (8–9 years) showed more clear positive associations between diet and sarcopenia features [25,26]. In addition, most existing studies focus on geriatric populations [19] or those with a disease [27].

Data regarding the relation between diet and muscle mass and/or the aSMI are scarce, possibly because young subjects are considered to have a low risk of developing sarcopenia. In fact, eating behavior has been related to the aSMI in young females [28], and Mediterranean diet adherence has been positively related to skeletal muscle mass in adolescents [29] and muscular fitness in Spanish university students [30]. Of note, it has been previously shown that more than 50% of young Japanese female university students with low physical activity had sarcopenia or tended to correspond to the aSMI cut-off for sarcopenia according to Asian criteria [31]. In addition, in a pilot study of 83 Japanese university students, 24.6% of females and 16.7% of males had pre-sarcopenia [32]. The prevalence of the disease in the elderly may increase up to 50% [8]. These alarming results suggest that the aSMI and its correlates should be further investigated, even in young subjects.

There is, thus, a need to clarify the role of nutrition, and the Mediterranean diet in particular, with skeletal muscle mass and related indices in young adults. Therefore, the aim of this pilot study was to investigate the relationship between Mediterranean diet adherence and the aSMI in a sample of healthy university students.

## 2. Materials and Methods

### 2.1. Study Design and Study Sample

**Figure 1:**
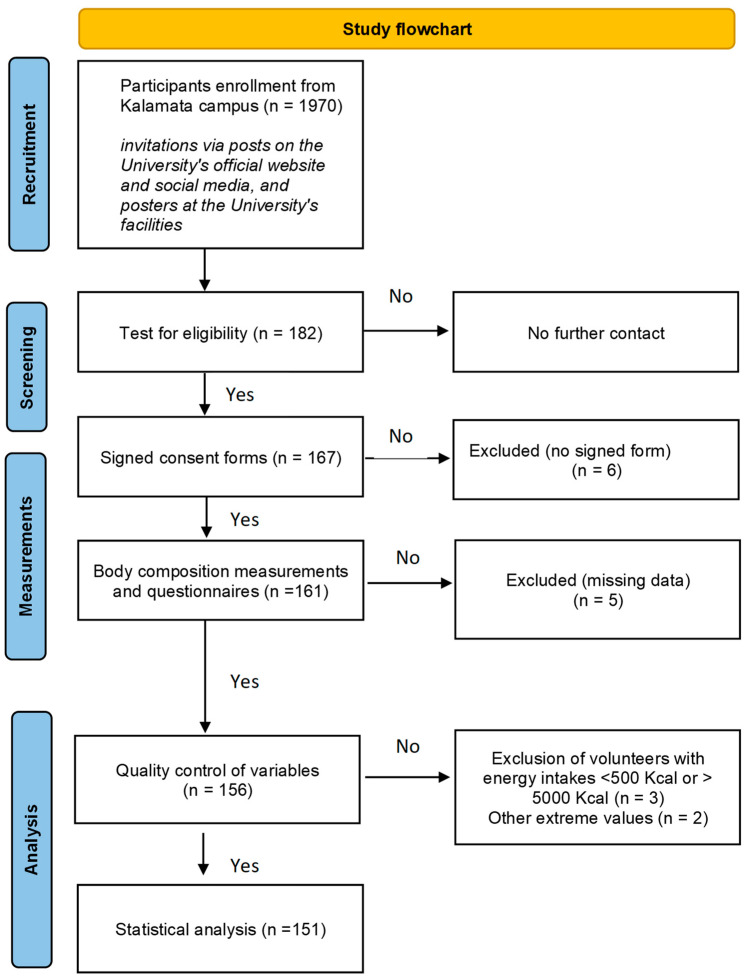
Flowchart of this study.

The present research was a pilot cross-sectional study. An easily accessible sample of undergraduate university students was recruited in June 2022 and January 2023. Students were invited via posts on the University’s official website and social media, as well as via written announcements (posters) at the University’s facilities. Several measurement days were planned, and students could choose the day best fitting their schedule. The eligibility criteria were (i) being a student of the University of the Peloponnese, (ii) having a good health status, and (iii) age 18–45 years. The exclusion criteria were (i) not being a student; (ii) not fulfilling the prerequisites for bioelectrical impedance analysis (BIA) measurement, i.e., refraining from strenuous exercise, caffeine, and alcohol for 8 h, fasting for 3 h prior to measurements (no foods, no liquids), and measurements in particular days in the menstrual cycle; (iii) pregnancy and lactation; (iv) alcohol and/or drug abuse; (v) the presence of medical conditions that may significantly impact skeletal muscle mass, such as myopathies, muscular dystrophy, and neuromuscular diseases; (vi) therapies related to fluid balance, such as diuretics; (vii) therapies related to muscle mass metabolism, such as chronic use of oral corticosteroids, growth hormone, etc.; (viii) orthopedic limitations that might interfere with accurate BIA measurements, such as artificial limbs or metal implants; and (ix) other limitations that might interfere with accurate BIA measurements, such as medical implants (pacemakers, patient monitoring systems) and insulin pumps. The flowchart of this study is shown in Figure 1.

### 2.2. Ethics

The research protocol was approved by the Ethics Committee of the School of Health Sciences of the University of the Peloponnese (protocol number 129, 6 July 2022), and all participants signed a consent form.

### 2.3. Evaluation of Dietary Habits and Diet Quality

A semi-quantitative Food Frequency Questionnaire (FFQ), validated for the Greek population, was used [33]. The FFQ included 69 questions concerning the frequency and serving size of dairy products (1 glass, 30 g cheese), eggs (1 item), starches (1 slice of bread), starchy vegetables (1/2 cup cooked), meat and poultry (60 g), fish (60 g), legumes (1/2 plate), vegetables (1/2 cup cooked or 1 cup raw), fruits (1 item), sweets (1 piece), alcohol (1 glass of various volumes according to alcohol content), stimulants, fats, and oil consumption [33].

The Mediterranean Diet Score (MedDietScore) was calculated using the subjects’ responses to the FFQ [34]. This score assesses adherence to the Mediterranean Diet by measuring the consumption of key food groups, with scores ranging from 0 to 55 (scores toward the upper scale indicate higher adherence) [34]. More specifically, firstly, the frequencies of food intakes were recoded to daily and monthly intakes. Then, foods were grouped according to the MedDietScore categories (non-refined cereals, potatoes, fruits, vegetables, legumes, fish, olive oil, red meat and products, poultry, full-fat dairy, and alcohol), and the intakes for each food group were calculated. It is noted that not all foods were included in the MedDietScore calculation, due to the score’s design. For example, refined cereals and low-fat dairy were not included. Portion adjustments were made if needed. The components of the MedDietScore were calculated, i.e., non-refined cereals score, potatoes score, fruit score, vegetable score, legumes score, fish score, olive oil score, red meat and products score, poultry score, full-fat dairy score, and alcohol score. For the consumption of foods close to the Mediterranean dietary pattern, scores of 0, 1, 2, 3, 4, and 5 were assigned for various frequencies, ranging from no consumption to daily consumption. Reverse scores were assigned for foods away from the Mediterranean dietary pattern. For alcohol, a score of 5 was considered for the consumption of less than 300 mL/day, a 0 score for the consumption of more than 700 mL/day or none, and scores 1–4 were considered for drinking 300–400, 400–500, 500–600, and 600–700 mL/day, respectively. The MedDietScore is derived from the sum of the food group scores [34].

Energy intakes lower than 500 Kcal or greater than 5000 Kcal were not considered plausible (but no subject had such intake). The USDA database was used to evaluate the energy and macronutrient content of foods [35]. The dietary content in carbohydrates, fats, and protein is shown in g/day, g/kg (for protein and carbohydrates) as well as in % of total energy.

### 2.4. Assessment of Physical Activity and Total Energy Requirements

Participants fulfilled the Hellenic Physical Activity Questionnaire (HPAQ) [36] for physical activity assessment. This questionnaire has been adapted to the habits of the Greek population [36]. Metabolic equivalents (METs) for each of the physical activities were calculated [36], and MET-min per week values were calculated as a measure of energy expenditure. One MET is the resting energy spent, and it equals 1 kcal/kg/h [37].

Resting energy requirements (REEs) were calculated by using available equations in the literature (Schofield) [38]. The physical activity level (PAL) was calculated as the ratio of total energy expenditure to resting energy requirements [37]. Total energy requirements were then calculated by multiplying the REE by the PAL for each individual [37].

### 2.5. Anthropometry and Body Composition

Body weight and height were measured with a scale (Tanita MC-780, Tokyo, Japan) and a stadiometer (Seca, Hamburg, Germany), respectively, to the nearest 0.1 kg for weight and the nearest 0.1 cm for height [39]. The BMI was determined as the ratio of weight divided by height squared, and the students were categorized as underweight, normal weight, or overweight/obese [40]. The measurements of waist and hip circumferences were performed with a tape (Seca, Hamburg, Germany) to the nearest 0.1 cm [41].

Body composition was assessed via multi-frequency BIA (Tanita MC-780, Japan) measuring at 5 kHz, 50 kHz, and 250 kHz [41]. In the present study, we have used measurements by the device at 50 kHz. The Tanita model used provides data on lean body mass (absolute quantity and % of total body weight), fat mass (absolute quantity and % of total body weight), intracellular water (absolute quantity, and % of total body water), extracellular water (absolute quantity and % of total body water), phase angle, aSMI, etc. It is noted that it provides total and segmental measurements (trunk, legs, arms). In the present analysis, we report the total measurements and aSMI, which is computed by taking into account the lean mass of the arms and legs and directly provided in the report form. For BIA measurements, a specific protocol was followed regarding hydration prerequisites and menstrual status, as previously described [42]. Subjects stepped with bare feet on the device, and the weight of their clothes was estimated and subtracted from the measured weight. Then, subjects held the electrodes, and the measurement was performed in a standing position [43]. Increased fat mass (as derived from BIA measurements) was assessed based on previously reported values [44]. More specifically, for females aged 20–39, the % body fat was considered increased if it was higher than 33%, and for females aged 40–59, the % body fat was considered increased if it was higher than 34%. For males aged 20–39, the % body fat was considered increased if it was higher than 20%, and for males aged 40–59, the % body fat was considered increased if it was higher than 22%.

Lastly, triceps skinfold was measured according to standard procedures [41], as an index of nutritional status [45]. All procedures were performed by two members of the technical staff of the Department of Nutrition and Dietetics.

### 2.6. Smoking Status

Smokers were classified as current smokers (smoking at least one cigarette per day, using tobacco or electronic cigarettes), non-smokers, and former smokers. The type of smoking was assessed. More specifically, subjects were asked to report whether they smoke cigarettes, tobacco, or electronic cigarettes. For current smokers, the number of cigarettes per day was recorded, as well as the total years of smoking.

### 2.7. Statistical Analysis

The Kolmogorov–Smirnoff criterion was used to assess whether variables were normally distributed. Variables with a normal distribution are presented as means ± standard deviation, while variables not following a normal distribution are presented as medians and 25th–75th percentiles. For categorical ones, frequencies were used (*n*, %). The t-test and Mann–Whitney test were used to test differences between men and women, for normally and non-normally distributed variables, respectively. The Chi-square test was applied to test for differences in frequencies between men and women. The Spearman correlation coefficient was used to test correlations between the aSMI, food groups, and MedDietScore. Simple and multiple linear regression models with log-aSMI as a dependent variable were constructed. The significance level was 5%. Statistical analysis was performed with the program IBM SPSS Statistics for Windows 29.0.0.0 241 (Armonk, NY, USA, IBM Corp.).

## 3. Results

### 3.1. Descriptive Characteristics of the Students

In total, 151 students participated (*n* = 114 females). Several characteristics of the participants are shown in Table 1. Overweight and obese subjects were grouped in the reporting. Females had higher levels of body fat compared to males, while males had higher levels of waist circumference, body water, and lean mass.

### 3.2. Dietary Intake of the Participants

The food intake of the participants is shown in Table 2. The MedDietScores were 29.0, 26.0–34.0, and 31.0, 27.0–34.0 (*p* = 0.172) in males and females, respectively (medians, interquartile ranges). No sex differences were documented in food group intake, except red meat.

### 3.3. Relation of aSMI with Food Group Intake

Spearman correlation coefficients were performed between the aSMI and food groups in a sex-specific analysis. In females, no significant association was found. In males, the aSMI was positively associated with fruit intake (Spearman’s rho = 0.418, *p* = 0.011), vegetable intake (Spearman’s rho-0.393, *p* = 0.018), fish intake (Spearman’s rho = 0.492, *p* = 0.002), and olive oil intake (Spearman’s rho = 0.305, *p* = 0.029).

### 3.4. Relation of aSMI with MedDietScore

**Figure 2:**
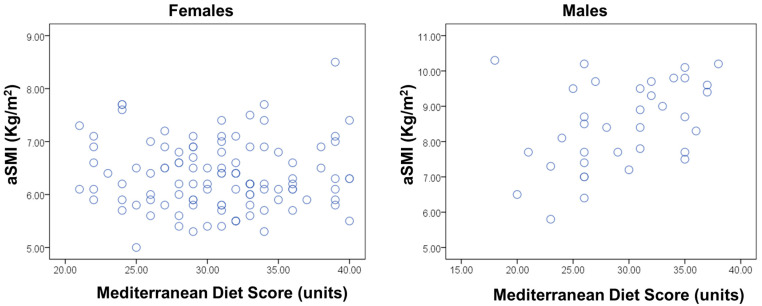
Scatter dot diagram for aSMI and MedDietScore in males and females.

Spearman correlation coefficients were performed between the aSMI and MedDietScore in a sex-specific analysis. In females, no significant association was found (Spearman rho = −0.036, *p* = 0.710). In males, a positive association was documented (Spearman rho = 0.416, *p* = 0.012) (Figure 2). Furthermore, linear regression models were applied, with log-aSMI as the dependent variable and MedDietScore as the independent variable after adjusting for basic covariates (Table 3). Multi-adjusted models were not tested in the sex-specific analysis due to the small sample size. In the total sample, the MedDietScore was also positively related to log-aSMI in linear regression models adjusted for age, sex, BMI, academic year, smoking, and physical activity (B = 0.009, SE = 0.004, *p* = 0.05). This model explained 71% of the total variance of log-aSMI (Table 4).

### 3.5. Relation of aSMI with Macronutrient Intake

Macronutrients were entered into the models one by one (to avoid multi-collinearity). From all models, only % fat was positively related to log-aSMI in men (B = 3.0 × 10^−5^, SE = 10^−5^, *p* = 0.027, 95% CI: 3.5 × 10^−6^; 5.8 × 10^−5^). No other significant relationship was identified.

## 4. Discussion

The present cross-sectional study assessed the relationship between diet and the aSMI in university students and revealed a positive relation between the MedDietScore and aSMI irrespective of various confounding factors.

Adherence to the Mediterranean diet has been positively associated with muscle mass in middle-aged and older adults [52,53,54]. Moreover, several systematic [23,24,55,56,57,58,59] and narrative reviews [18,60,61,62] have investigated the relationship between several dietary patterns and sarcopenia. Our group has also reviewed the relationship between the Mediterranean diet and sarcopenia in older adults [19]. Concerning the aSMI, a healthy dietary pattern has been associated with the aSMI in the general population [63] and older adults [64]. No relation was found between Mediterranean diet adherence and fat mass in adults with overweight and obesity [65], while in the same study, the alternate Mediterranean diet score was positively associated with physical activity, suggesting higher levels of muscle functionality [65]. Previous studies have also found that the adoption of the Mediterranean diet is associated with better gait speed, body support, and hand-grip strength and a lower risk of disability [19,20,52,66]. Thus, Mediterranean diet adherence seems to have a myo-protective effect [60]. Indeed, in Mediterranean diet-based programs targeting weight loss, a low extent of lean tissue loss has been documented [67,68].

The aSMI constitutes a clinically applicable definition of sarcopenia [11,12]. Low values of the aSMI indicate sarcopenia presence, which may have resulted from increasing age, sedentary lifestyle, endocrine problems, disease states, subclinical inflammation, and malnutrition [11]. In parallel, skeletal muscle is associated with cardiometabolic health [2] and mortality [69] and decreases with age [70]. The aSMI measured in our study aligns with the findings previously reported for young adults from Lebanon [71]. The median value of the aSMI was well above the threshold value indicative of sarcopenia (6.75–7.40 kg/m^2^ for males and 5.07–5.80 kg/m^2^ for females) [47], although several population-specific cutoff points have been suggested [72,73]. Of note, it has been previously shown that more than 50% of young Japanese female university students with low physical activity had or tended to have sarcopenia [31]. In addition, a pilot study showed that 24.6% of females and 16.7% of males had pre-sarcopenia [32]. However, there are few studies assessing the relation of diet and other lifestyle variables with the aSMI in young subjects. In this context, the aSMI has been related to gait speed [74], exercise habits [75], normal-weight obesity [76], and eating behavior [28] in young females. Mediterranean diet adherence has been also positively related to skeletal muscle mass in Chinese adolescents [29] and fitness in Spanish university students [30].

The median MedDietScore in the present study was 31, which could be considered relatively low in the range of 0–55. The MedDietScore was first validated by data from the ATTICA study, which was conducted in healthy Greek adults [34]. In that study, the median MedDietScore was 26.5 (first tertile 27.0, third tertile 32.0) [34], similar to values reported in the present study i.e., median 31 (first tertile 27.0, third tertile 34.0). Similarly, in a recent study by our group conducted on Greek university students, the median, first, and third tertile of the MedDietScore were 29, 24.5, and 32 for men and 30, 27.0, and 34.0 for females, respectively, suggesting a similar distribution pattern of MedDietScore values [77]. Even though the third tertile cutoff was only 32 in the ATTICA study [34] vs. 34 in the present study, several results have suggested the protective effects of Mediterranean diet adherence at these levels. Indeed, subjects in the third vs. first tertile of the MedDietScore had lower inflammatory and coagulation markers [78], CVD, and related diseases [79]. It is thus shown that the MedDietScore may be relatively low from a numerical perspective but has substantial effects from a clinical perspective. Similarly, other scores of Mediterranean diet adherence, have also been “validated” against cardiovascular outcomes, even at relatively low absolute scores. For example, in the study of Stefler et al., a higher adherence to the Mediterranean diet was associated with a reduced risk of CVD [80]. This non-Mediterranean population had a relatively low adherence to the Mediterranean diet (one-quarter of the study sample scored more than ten points, corresponding to about 60% of the maximum score). It seems that the rule “the higher the better” applies, even at low absolute scores. Given the previously reported beneficial effects of the MedDietScore, the present results may provide an additional mechanism through which the Mediterranean diet exerts its beneficial effects.

The positive association of the Mediterranean diet with the aSMI suggests its importance in maintaining/increasing muscle mass in young males, possibly through its content of proteins of plant and animal origin, as well as its antioxidant and anti-inflammatory substances. In a nutrient-based approach, the Mediterranean diet is rich in antioxidants, anti-inflammatory substances, fiber, monounsaturated fatty acids, and omega-3 fatty acids [81]. Indeed, muscle loss may take place in inflammatory and oxidative stress states [82]. In this context, adopting a healthy, “anti-inflammatory diet” may be useful for subjects with low muscle mass [8]. For example, in older subjects, low selenium, vitamin C, and E levels are related to reduced muscle mass [83], while high levels of carotenoids have been associated with lower disability risk [83]. Omega-3 fatty acids also exert anti-inflammatory actions [84], while saturated fatty acids have the opposite effect [85]. Interestingly, in the present study, the fat content of the diet (% of energy) was related to the aSMI, underlying the importance of healthy fats and fat-soluble vitamins in muscle tissue. In a food-based approach, the Mediterranean diet includes fruits, vegetables, fish, olive oil, legumes, whole grains, nuts, and seeds; a moderate intake of dairy products and wine consumption; and limits the consumption of processed foods, sweets, and red meat [86]. Several studies have also shown favorable associations between sarcopenia and the intake of fish oil [87], fruits, [88], and vegetables [88], which are in line with the associations observed in this study. Moreover, the adoption of the Mediterranean diet is related to a lower incidence of diseases, such as diabetes [89] and cancer [90], and lower rates of frailty [91], which may be related to muscle health.

In this study, the association between the MedDietScore and muscle mass was evident in males. Similarly, in the study of Tian et al., Mediterranean diet adherence (as assessed with a different index) showed positive associations with muscle indices in male, young subjects [54]. Moreover, the relationship between fruits and vegetables and sarcopenia was most evident in older males in an epidemiological study in Korea [88]. Males may also be more sensitive to muscle mass changes. In particular, longitudinal changes in fat-free mass are greater in males compared to females [92]. In parallel, reductions in appendicular lean mass are greater in males compared to females [93,94]. Myostatin increases in cases of sarcopenia mostly in females, while in males, it serves as a homeostatic regulator of muscle mass, suggesting sex differences in muscle physiology [95]. It is also noteworthy that more than 3000 genes are differentially expressed in skeletal muscle between males and females [96]. Sex hormones affect muscle growth and fiber size [97], while other hormones, such as thyroid ones, may differentially act in males and females [97]. Epigenetic sex differentiations have been also documented in response to training [98].

Along with the presentation of our results, some limitations should be reported. This study was cross-sectional, and causal relationships cannot be substantiated. In addition, the sample consisted exclusively of students from a single university. A convenience sampling method was followed, so the sample cannot be considered representative. Most participants were students in the Department of Nutrition, which may potentially influence their dietary choices and reported consumption [99]. The females’ participation outweighed that of the males. Since most students were studying Dietetics, this imbalance may reflect the gender differences observed in registered dietitians [100]. These factors limit the generalizability of the present findings. Another issue is the small number of male participants, in whom significant results were documented. The observed power for MedDietScore tertiles in men was 50–59% (post hoc analysis). However, our results can be considered as pilot ones and may encourage researchers to conduct a larger study with more men to test the present results. In addition, according to the results of Lampropoulou et al. [101], a sample size of 161 is required to achieve 75% power to investigate the relationship between the Mediterranean diet and lean mass (G-POWER 3.1.0.7). Regarding dietary assessment, a self-administered FFQ was used, and a possibility of under- or over-reporting may be present. Lastly, the aSMI was measured with BIA, while other more reliable methods exist. Whole-body magnetic resonance imaging (MRI) constitutes the gold standard for the assessment of skeletal muscle [102], while DXA and CT have also been used to assess the aSMI [11]. Several factors may influence BIA measurements, but a strict protocol was followed to minimize potential errors [43].

## 5. Conclusions

In conclusion, the present pilot study supports the importance of the adoption of a Mediterranean dietary pattern—rich in plant proteins and antioxidant/anti-inflammatory compounds—in maintaining/attaining muscle mass in young subjects. Future clinical studies are needed to confirm this relationship and clarify the possible explanatory mechanisms.

## Figures and Tables

**Table 1:** Descriptive characteristics of participants.

	Total	Males	Normal Value *	*p*-Value §	Females	Normal Value *	*p*-Value §	*p*-Value ‡
Number of participants (*n*)	151	37			114			
Age (years)	20.88 ± 4.1	20.89 ± 4.35	NA	NA	20.88 ± 4.04	NA	NA	0.783
Year of studies								
*1st (n)*	45	14	NA	NA	31	NA	NA	0.300
*2nd (n)*	47	15	NA	NA	32	NA	NA	0.200
*3rd (n)*	44	4	NA	NA	40	NA	NA	0.004
*4th (n)*	12	4	NA	NA	8	NA	NA	0.500
Current smoking (*n*)	30	7	NA	NA	23	NA	NA	0.538
Years of smoking	4 (2.75–6.25)	6 (4–6)	NA	NA	3 (2–5)	NA	NA	0.124
Cigarettes per day (number)	5 (7–12.5)	10 (7–10)	NA	NA	5.5 (4–10.2)	NA	NA	0.152
BMI (kg/m^2^)	22.0 ± 2.9	22.8 ± 3.1	18.5–25		21.7 ± 2.8	18.5–25		0.040
*Underweight (n)*	15	2			13			0.300
*Normal weight (n)*	115	26			89			0.300
*Overweight and Obese (n)*	21	9			12			0.030
Lean body mass (kg)	44.7 (40.6–51.6)	63.2 (55.5–67.1)	NA **	NA	42.4 (40.2–46.2)	NA **	NA	<0.001
Fat mass (%)	23.0 ± 7.6	15.65 ± 7.05			25.51 ± 6.09			<0.001
Increased fat mass (*n*) †	21	9			12			0.056
Fat mass (kg)	14.2 ± 5.6	11.5± 5.4	NA **	NA	15.2 ± 5.4	NA **	NA	<0.001
Intracellular water (kg)	17.3 (15.9–19.5)	25.9 (23.4–28.2)	NA **	NA	16.7 (15.6–17.7)	NA **	NA	0.024
Extracellular water (kg)	13.2 (12.2–15.6)	17.4 (16.2–18)	NA**	NA	12.7 (12.1–13.6)	NA **	NA	<0.001
aSMI (kg/m^2^)	6.50 (6.0–7.40)	8.45 (7.55–9.57)	>6.75–7.40	<0.001	6.20 (5.90–6.80)	>5.07–5.80	<0.001	<0.001
PhA (°)	5.5 (5.1–6.4)	6.8 (6.1–7.3)	8.0 (supine position)	<0.001	5.3 (5.1–5.9)	7.0 (supine position)	<0.001	<0.001
Wrist Circumference (cm)	15.5 (14.6–16.4)	17.0 (16.3–18.0)	NA	NA	15.0 (14.5–16.0)	NA	NA	<0.001
Waist Circumference (cm)	71.0 (67.0–75.0)	78.0 (74.0–81.0)	<102	<0.001	69.0 (66.0–72.0)	<88	<0.001	<0.001
Hip Circumference (cm)	97.0 (93.0–101.0)	98.0 (95.0–103.0)	NA ***	NA	97.0 (92.0–101.0)	NA ***	NA	0.057
Triceps skinfold (mm)	14.9 (12.0–20.0)	11.0 (8.8–15.1)	12.0 (mean), >4.5 (5th percentile)	0.382, <0.001	15.6 (13.3–20.3)	23 (mean) >11.0 (5th percentile)	<0.001, <0.001	<0.001
Physical activity								
Total MET/minute/day	1832 (1689–2034)	1844 (1741.5–2196.5)	NA	NA	1821.5 (1682.5–1996.5)	NA	NA	0.277
Estimated total energy requirements (Kcal/day)	1920 (1647–2179)	2229 (1960–3054)	NA	NA	1827 (1603–2078)	NA	NA	<0.001
Sleep duration (hours)	7.0 (6.0–8.0)	7.5 (6.2–8.0)	NA	NA	7.0 (6.0–8.0)	NA	NA	0.899
Screen time			NA	NA		NA	NA	
Time watching TV/video (hours)	3.0 (2.0–4.0)	2.0 (2.0–3.5)	NA	NA	3.0 (2.0–4.0)	NA	NA	0.288
Time on the computer (hours)	5.0 (2.0–12.0)	5.0 (3.0–12.0)	NA	NA	5.5 (2.0–10.5)	NA	NA	0.727

Data are presented as means ± standard deviation for normally distributed variables or as median and interquartile range for non-normally distributed variables. Categorical variables are displayed as *n* (%). *t*-test (for normal variables) or Mann–Whitney test (for non-normal variables) was used to compare values between males and females. The chi-square test (for categorical variables) was used to compare categorical variables between males and females. † Increased fat mass corresponding to obesity was based on previously reported values [44]. More specifically, for females aged 20–39, the % body fat was considered increased if it was higher than 33%, and for females aged 40–59, the % body fat was considered increased if it was higher than 34%. For males aged 20–39, the % body fat was considered increased if it was higher than 20%, and for males aged 40–59, the % body fat was considered increased if it was higher than 22%. § Comparison of values with gender-specific reference values. ‡ Comparison of values between males and females. * The categorization of students as underweight, normal weight, or overweight/obese was based on the criteria of the World Health Association [40]. Normal values for % body fat were based on data from Kyle et al., who included measurements of bioelectrical impedance analysis [46]. For the aSMI, values lower than 6.75–7.40 kg/m^2^ for males and 5.07–5.80 kg/m^2^ for females are indicative of sarcopenia [47]. Reference values for waist circumference were based on the American Heart Association/National Heart, Lung, and Blood Institute [48]. Reference values for triceps skinfold were based on data from Bishop et al. [49]. Reference values for PhA were based on data from Barbosa-Silva et al. [50], measured in the supine position. Since reference values are sex- and age-specific, a mean value was considered. More specifically, for young males 18–20 y, 20–29 y, and 30–39 y, a mean PhA ± SD of 7.90 ± 0.47, 8.02 ± 0.75, and 8.01 ± 0.85 has been reported, and the authors have set the normal value at 8.0°. For young females 18–20 y, 20–29 y, and 30–39 y, a mean PhA ± SD of 7.04 ± 0.85, 6.98 ± 0.92, and 6.87 ± 0.84° has been reported, and the authors have set the normal value at 8.0^o^. It is noted that the existing reference values were based on measurements taken in a supine position. However, PhA is lower in a standing position (~1° lower) [51]. ** Normal values cannot be calculated for these variables as they depend on total body weight. *** Normal values cannot be calculated for these variables. There are normal values only for the waist-to-hip circumference ratio. aSMI: appendicular skeletal muscle index; BMI: Body mass index; MET: Metabolic equivalents; NA: Not applicable or not available; PhA: Phase angle.

**Table 2:** Dietary intake of participants.

Parameters Evaluated	Total Sample (*n* = 151)	Males (*n* = 37)	Females (*n* = 114)	*p*-Value
Refined cereals (servings per day)	2.18 (1.41–3.48)	1.62 (1.26–3.48)	2.18 (1.55–3.50)	0.267
Unrefined cereals (servings per day)	0.64 (0.19–1.42)	0.62 (0.09–1.42)	0.64 (0.19–1.44)	0.369
Potatoes (servings per day)	0.26 (0.11–0.36)	0.26 (0.18–0.68)	0.21 (0.11–0.36)	0.108
Fruit (servings per day)	1.77 (0.83–3.20)	1.71 (0.94–3.09)	1.77 (0.77–3.22)	0.943
Vegetables (servings per day)	1.95 (0.95–2.77)	1.47 (0.75–2.47)	2.01 (1.02–2.79)	0.055
Legumes (servings per day)	0.53 (0.16–0.53)	0.53 (0.16–0.53)	0.53 (0.16–0.53)	0.388
Fish (servings per day)	0.33 (0.16–0.53)	0.33 (0.00–0.53)	0.33 (0.16–0.53)	0.577
Red meat (servings per day)	0.99 (0.56–1.80)	1.61 (0.91–2.34)	0.90 (0.49–1.65)	0.002
Poultry (servings per day)	0.53 (0.53–1.60)	0.53 (0.53–1.60)	0.53 (0.53–1.60)	0.764
Eggs (servings per day)	0.21 (0.21–0.64)	0.21 (0.21–0.64)	0.42 (0.21–0.64)	0.925
Full-fat dairy (servings per day)	0.64 (0.21–1.00)	0.64 (0.13–1.14)	0.64 (0.21–0.85)	0.540
Low-fat dairy (servings per day)	0.28 (0.06–1.00)	0.21 (0.06–0.85)	0.53 (0.06–1.00)	0.388
Sweets/Treats (servings per day)	0.56 (0.26–0.98)	0.47 (0.27–0.84)	0.56 (0.26–1.05)	0.571
Chips/Popcorn (servings per day)	0.06 (0.00–0.21)	0.06 (0.00–0.14)	0.06 (0.00–0.21)	0.737
Alcohol (servings per day)	0.21 (0.06–0.49)	0.19 (0.06–0.49)	0.24 (0.06–0.49)	0.896
Energy intake (Kcal per day)	1920 (1647–2179)	2229 (1960–3054)	1827 (1603–2078)	<0.001
MedDietScore	31.0 (26.0–34.0)	29.0 (26.0–34.0)	31.0 (27.0–34.00)	0.172
Fat (g/day)	130 (106–173)	129 (98–194)	133 (109–170)	0.896
Fat (% energy)	51.5 ± 7.0	50.0 ± 8.1	52.1 ± 6.5	0.120
Carbohydrates (g/day)	197 (145–261)	211 (143–304)	193 (149–258)	0.408
Carbohydrates (g/kg)	3.3 (2.3–4.3)	3.0 (2.1–4.2)	3.5 (2.4–4.6)	0.144
Carbohydrates (% energy)	33.7 ± 6.4	34.1 ± 7.0	33.6 ± 6.2	0.687
Proteins (g/day)	88 (64–117)	101 (65–139)	82 (63–114)	0.008
Proteins (g/kg)	1.4 (1.0–2.0)	1.4 (0.9–1.9)	1.4 (1.0–2.0)	0.546
Proteins (% energy)	14.6 ± 2.7	15.9 ± 2.9	14.3 ± 2.5	0.002

Data are presented as means ± standard deviation for normally distributed variables or as median and interquartile range for non-normally distributed variables. Categorical variables are displayed as *n* (%). The Mann–Whitney test (for non-normal variables) was used to compare values between males and females. The chi-square test (for categorical variables) was used to compare categorical variables between males and females.

**Table 3:** Linear regression models with log-appendicular skeletal muscle index (aSMI) as dependent variable in males and females.

	Age-BMI Adjusted Model							
	Males				Females			
	R^2^ = 37.1%				R^2^ = 39.4%			
	B	SE	95% CI	*p*	B	SE	95% CI	*p*
Age (years)	0.001	0.002	−0.004; 0.005	0.824	0.001	0.001	−0.01; 0.002	0.355
BMI (kg/m^2^)	**0.009**	**0.003**	**0.003; 0.015**	**0.005**	**0.010**	**0.001**	**0.007; 0.012**	**<0.001**
MedDietScore (3rd vs. 1st tertile)	**0.027**	**0.011**	**0.004; 0.050**	**0.023**	**−0.0004**	0.004	−0.009; 0.008	0.920

Bold denotes that variables significantly predict the dependent variable “a-SMI”. aSMI: appendicular skeletal muscle index; BMI: body mass index; CI: confidence interval; MedDietScore: Mediterranean diet score.

**Table 4:** Multi-adjusted linear regression models with log-appendicular skeletal muscle index (log aSMI) as the dependent variable in the total sample.

	Total Sample, *n* = 151			
	R^2^ = 71%			
	B	SE	95% CI	*p*
Sex (males = 1)	0.111	0.008	0.096; 0.127	<0.001
Age (years)	0.001	0.001	−0.001; 0.003	0.316
BMI (kg/m^2^)	0.009	0.001	**0.007; 0.012**	**<0.001**
Smoking (1 = yes)	−0.002	0.009	−0.019; 0.015	0.809
ΜΕΤ-min/day	18 × 10^−6^	6.7 × 10^−6^	**4.5 × 10^−6^; 3.1 × 10^−6^**	**0.009**
Academic year (1–4)	−0.001	0.004	−0.009; 0.006	0.762
MedDietScore (3rd vs. 1st tertile)	0.009	0.004	**−6 × 10^−5^; 0.017**	**0.05**

Bold denotes that variables significantly predict the dependent variable “a-SMI”. aSMI: appendicular skeletal muscle index; BMI: body mass index; CI: confidence interval; MedDietScore: Mediterranean diet score.

## Data Availability

The data presented in this study are available on request from the corresponding author.
